# The effects of composite photosynthetic bacterial inoculant PS21 on the biochemical characteristics of wheat seedlings under tetrabromobisphenol A stress

**DOI:** 10.1080/13102818.2014.999298

**Published:** 2015-01-13

**Authors:** Hong-Lian Ge, Fu-Li Zhang

**Affiliations:** ^a^ College of Life Science and Agronomy, Zhoukou Normal University, Zhoukou 466001, China

**Keywords:** bacterium inoculants, tetrabromobisphenol A, chlorophyll content, soluble sugar, soluble protein, wheat seedling, antioxidant enzymes

## Abstract

The aim of this study was to investigate whether the composite photosynthetic bacterial inoculant PS21 alleviate the damage inflicted on wheat seedlings by tetrabromobisphenol A (TBBPA). The biochemical characteristics of wheat seedlings were analysed through laboratory simulation after co-treating seedlings with PS21 and 0, 5, 10, 20, 40, 80 and 100 mg kg^−1^ TBBPA, respectively. The results showed that TBBPA reduced the total chlorophyll content and increased the malondialdehyde (MDA) content. TBBPA increased the soluble sugar content, soluble protein content and activate superoxide dismutase (SOD; EC: 1.15.1.1), catalase (CAT; EC: 1.11.1.6) and peroxidase (POD; EC: 1.11.1.7) at low concentrations, while it reduced soluble sugar content, soluble protein content and decreased the activities of SOD, CAT and POD at high concentrations. At the concentration of 10^7^ CFU mL^−1^, PS21 could markedly relieve the toxicity of different concentrations of TBBPA on wheat seedlings. Wheat seedlings treated with both TBBPA and PS21 showed a higher soluble sugar content, higher soluble protein content, higher SOD, CAT and POD activities, and a lower MDA content as compared to those treated only with TBBPA. The composite photosynthetic bacterial inoculant PS21 significantly alleviates the damage inflicted on wheat seedlings by TBBPA.

## Introduction

Tetrabromobisphenol A (TBBPA) is currently the most produced and used brominated flame retardant, and is widely used in electronic equipment, plastics and textiles.[1–3] TBBPA is frequently released into the environment during the production, consumption and disposal of TBBPA-containing products.[4,5] TBBPA has been detected in air, soil, and sewage samples, sludge sediment, plants and animals.[6–11] TBBPA exhibits high toxicity on aquatic animals, mammals and humans. It enters and accumulates in humans through the food chain, as long-term exposure of TBBPA may impair brain and bone development, and may even cause cancer.[12] Moreover, it has been found to have immune toxicity and cytotoxicity and interferes with the functioning of the endocrine system.[13–18] Studies have shown that TBBPA can reduce growth, change metabolism and damage the cell membrane and antioxidant enzymes in plants.[19,20] The deleterious effects of TBBPA on ecosystems are causing increasing concerns. Most TBBPA studies were focused on the detection, degradation and toxicity of this chemical, while studies to find ways to mitigate TBBPA-caused damage on crops have rarely been reported.

Purple photosynthetic bacteria are a type of phototrophic bacteria that are capable of carbon sequestration, nitrogen fixation and degradation of organic chemicals; however, they do not produce oxygen, and some strains can also secrete antiviral factors and growth hormones.[21–24] Purple photosynthetic bacteria have been widely used as biological fertilizers in agriculture that can increase chlorophyll content of seedlings, enhance photosynthesis and promote the growth of plant roots.[25–27] Studies show that certain concentrations of purple photosynthetic bacteria can mitigate the toxic effects of heavy metals, UV, or salt stress on seedling growth and antioxidant enzymes, and increase stress resistance of plant seedlings.[28–30] Nevertheless, whether the composite inoculants of purple photosynthetic bacteria can relieve the toxicity of TBBPA is still unknown. Using wheat as a model, we showed that the inoculants of purple photosynthetic bacterium PS21 were able to alleviate the toxic effects of TBBPA on wheat seedlings. Furthermore, we attempted to understand the underlying mechanism of the detoxification of PS21. Thus, our study will enable the further development and application of composite photosynthetic bacterium inoculants.

## Materials and methods

### The soil

Soil was collected from the experimental field of Zhoukou Normal University, and only the uncultivated and uncontaminated topsoil (0–20 cm) was used. The basic physical and chemical properties of the soil used were as follows: soil texture was sandy loam, soil organic matter content was 15.8 g kg^−1^, soil pH value 7.5, soil alkali-hydrolyzable nitrogen 65.3 mgkg^−1^, available potassium 120.0 mgkg^−1^ and available phosphorus 21.4 mg kg^−1^. The soil was sifted through a 2 mm sieve, autoclaved at 101 kPa, 121 °C and dried.

### Microbial inoculants

The composite photosynthetic bacterium inoculant PS21, consisting of the stress resistant and growth-promoting photosynthetic bacterial (PSB) strains AR12 and AR13, was developed in our laboratory. The two strains were identified as Rhodopseudomonas *sp. and* Rhodospirillum sp., respectively. The PSB medium consisted of minimal medium supplemented with 0.15% yeast extract.[31] TBBPA used in the study was purchased from the Aladdin Chemistry Co. Ltd in Fengxian district, Shanghai, China. The cultivar of wheat used was Zhoumai18, provided by Zhoukou City Academy of Agricultural Sciences.

### Generation of composite photosynthetic bacterium inoculants

The PSB strains AR12 and AR13 were inoculated in the liquid PSB medium and cultured for 7 days under anaerobic conditions at 28 °C, with photosynthetic photon flux (PPF) of 100 μmoL m^−2^ s^−1^ light, and centrifuged (5000 × g, 50 min). Bacterial concentration was measured by a spectrophotometer at 660 nm wavelength; the concentration of bacteria was diluted to 10^9^ CFU mL^−1^ using sterile water. Equal volumes of the two photosynthetic bacteria were mixed to make the inoculant PS21, and the final titer was adjusted to 10^7^ CFU mL^−1^.

### Experimental design

The wheat seeds were surface sterilized in 70% ethanol for 1--2 min followed by 0.05% sodium hypochlorite for 10 min, and then rinsed in sterile water. The seeds were then placed on a sterilized filter paper within a Petri dish; after a small amount of distilled water was added onto the dish, the seeds were kept in an incubator at 25 °C in the dark for 48 h to induce germination. Air-dried soil samples were packed into pots (pots height 20 cm, diameter 25 cm). The pots were randomized into two groups namely group A and group B, which were further divided into seven subgroups. Soil samples of group A were sprayed with different concentrations of TBBPA (0, 5, 10, 20, 40, 80 and 100 mg kg^−1^ dry weight soil); soil samples of group B were treated similar to those of group ‘A,’ treatment except that 25 mL of composite photosynthetic bacterium inoculants PS21 was added. Each concentration of TBBPA was tested in three replicates and each replicate involved 10 pots. Wheat seedlings without TBBPA and PS21 treatment served as controls. Wheat seedlings were placed in an artificial climate chamber with illumination time as 12 h/day, day and night temperature as 30/22 °C, and relative humidity set to 70%. Physiological and biochemical parameters of leaves were determined at the later seedling stage (20 days after treatment) using leaves on the same position in the stem.

### Determination of physiological and biochemical parameters of wheat

Superoxide dismutase (SOD) activity was measured by Nitro blue tetrazolium chloride test and expressed as U mg^−1^ Pro;[32] catalase (CAT) activity was measured by the UV absorption method and reported as U mg^−1^ Pro;[32] peroxidase (POD) activity was measured using the guaiacol assay and reported as U mg^−1^ Pro;[32] chlorophyll content was determined by spectrophotometry using acetone and 95% ethanol (V:V = 1:1) extracts, reported as mg g^−1^ FW;[33] malondialdehyde (MDA) content was determined by thiobarbituric acid assay, reported as μmol g^−1^ Pro;[33] soluble sugar content was determined by anthrone assay, shown as mg g^−1^ FW;[34] soluble protein content was determined by Coomassie brilliant method, shown as mg g^−1^ FW.[35]

### Data analysis

All data were the average of three replicates. The mean of the data and standard deviation (±SD) were calculated using Microsoft Excel 2003, and significant differences were analysed using the SPSS16.0 statistical analysis software.

## Results and discussion

### The effects of combined PS21 and TBBPA treatment on total chlorophyll content of wheat seedlings

**Figure 1.**
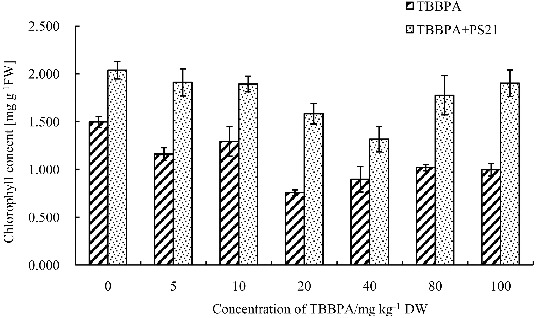
Changes of the total chlorophyll content in wheat leaves with combined treatment of PS21 and different concentrations of TBBPA. Values are mean ± SD and bars indicate standard deviation. TBBPA: treatment with various concentrations TBBPA (0–100 mg kg^−1^ DW); TBBPA + PS21: with combined treatment with PS21 and various concentrations TBBPA (0–100 mg kg^−1^ DW).

Chlorophyll is the main pigment involved in photosynthesis of plants, and its content reflects the physiological state of the leaves. Li et al. [20] suggested that the chlorophyll content can be used as a key indicator of the damage on plant growth and development caused by soil organics and heavy metals. Lee et al. [26] reported that *Rhodopseudomonas* sp. KL9 and BL6 strains can produce indole-3-acetic acid (IAA) and ALA (5-aminolevulinic acid) to regulate the metabolism of plants and promote plant growth. In order to analyse whether PS21 treatment could decrease the damage inflicted on wheat seedling by TBBPA, total chlorophyll content in wheat leaves were measured after PS21 treatment. As can be seen from Figure 1, when the TBBPA concentration was 20 mg kg^−1^, the total chlorophyll content of the wheat seedlings reached the lowest level, and increasing the concentration to 40 mg kg^−1^ did not lead to further decrease in the total chlorophyll content (*P* > 0.05). Compared to the control, the wheat seedlings treated with six concentrations of TBBPA varying from 5 to 100 mg kg^−1^ showed a marked decrease in the total chlorophyll content, and the total chlorophyll content was reduced by 22.45%, 13.57%, 49.47%, 40.13%, 31.96% and 33.41%, respectively (*P* < 0.05). There was no significant difference in the total chlorophyll content after treatment with 80–100 mg kg^−1^ TBBPA (*P* > 0.05). The total chlorophyll content increased significantly in all groups after the treatment of PS21 (*P* < 0.01). The wheat seedlings treated with different concentrations of TBBPA showed different degrees of alleviation of total chlorophyll reduction in response to PS21; however, a clear trend was not observed. The total chlorophyll content was increased by 64.56%, 46.37%, 109.27%, 46.88%, 74.35% and 90.74% in response to PS21 in wheat seedlings treated with 5, 10, 20, 40, 80 and 100 mg kg^−1^ TBBPA. PS21 showed the best effects on wheat seedlings treated with 20 mg kg^−1^ TBBPA in terms of increasing total chlorophyll content. In brief, our results are consistent with the findings of Lee et al., since PS21 treatment increased total chlorophyll content of wheat seedlings compared to control under TBBPA stress. Composite photosynthetic bacterium inoculants produce physiologically active substances to alleviate or abolish the toxic effects of TBBPA on chlorophyll synthesis and improve the total chlorophyll content of wheat seedlings.[25–27]

### The effects of combined PS21 and TBBPA treatment on soluble sugar content of wheat seedlings

**Figure 2.**
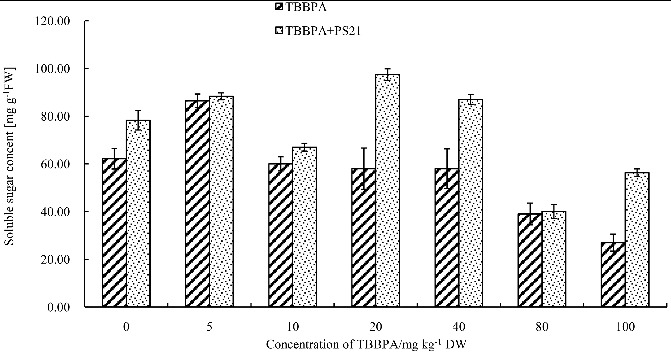
Changes of the soluble sugar content in wheat leaves with combined treatment of PS21 and different concentrations of TBBPA. Values are mean ± SD and bars indicate standard deviation. TBBPA: treatment with various concentrations TBBPA (0–100 mg kg^−1^ DW); TBBPA + PS21: with combined treatment with PS21 and various concentrations TBBPA (0–100 mg kg^−1^ DW).

Carbohydrates regulate plant growth, activate plant immune function, enhance disease resistance, and plants often accumulate some soluble sugars to reduce the freezing point and osmotic potential in order to cope with changes in the environment.[36,37] Therefore, the accumulation of soluble sugars can be also considered as a self-protective response against TBBPA. Our results showed that an increase in TBBPA concentration resulted in an increase in soluble sugar content of wheat seedlings at first followed by a decrease in content (Figure 2). The soluble sugar content reached the highest level when the TBBPA concentration was 5 mg kg^−1^ (increased by 38.71% compared to the control). Statistical analysis showed that, except 10, 20 and 40 mg kg^−1^ TBBPA, all of the other concentrations led to significant changes in soluble sugar content compared to the control (*P* < 0.05). A significant reduction (56.62%) in soluble sugar content of wheat seedlings was observed as a result of 100 mg kg^−1^ TBBPA application. Our results were in accordance with previous studies. Certain concentrations of TBBPA caused the plant cells to adjust their osmotic potential via soluble sugar accumulation to prevent membrane damage and to improve the water retention; thus, stabilizing the cell structure and preventing cell dehydration. However, high concentrations of TBBPA inhibit generation of soluble sugars and impair the stress resistance of plants.[36,37] After the treatment of PS21, soluble sugar content of wheat seedlings was significantly elevated (*P* < 0.05). Thus, PS21 can significantly alleviate the inhibition of soluble sugar synthesis by TBBPA; however, the effects varied without significant correlation with TBBPA concentration. PS21 had the best effects on wheat seedlings treated with 100 mg kg^−1^ TBBPA in terms of soluble sugar content, which increased by 108.67% in comparison to that of the seedlings only treated with 100 mg kg^−1^ TBBPA. Thus, the composite photosynthetic bacterium inoculants can alleviate the damage of TBBPA on wheat seedlings and improve the TBBPA resistance of wheat seedlings. In short, after the treatment of PS21, soluble sugar content of wheat seedlings increased significantly, improving plant stress resistance and the ability of plants to adapt to the environment.

### Effects of combined PS21 and TBBPA treatment on activities of antioxidant enzymes of wheat seedlings

**Figure 3.**
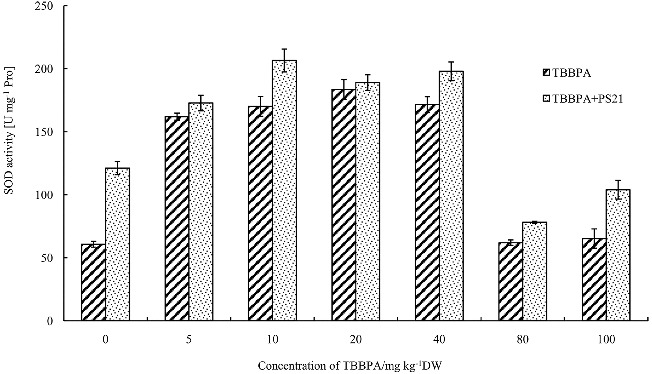
Changes of the SOD activity in wheat leaves with combined treatment of PS21 and different concentrations of TBBPA. Values are mean ± SD and bars indicate standard deviation. TBBPA: treatment with various concentrations TBBPA (0–100 mg kg^−1^ DW); TBBPA + PS21: with combined treatment with PS21 and various concentrations TBBPA (0–100 mg kg^−1^DW).

**Figure 4.**
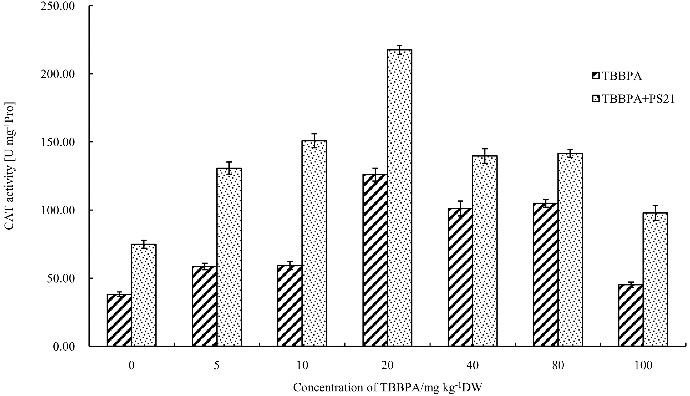
Changes of the CAT activity in wheat leaves with combined treatment of PS21 and different concentrations of TBBPA. Values are mean ± SD and bars indicate standard deviation. TBBPA: treatment with various concentrations TBBPA (0–100 mg kg^−1^ DW); TBBPA + PS21: with combined treatment with PS21 and various concentrations TBBPA (0–100 mg kg^−1^ DW).

**Figure 5.**
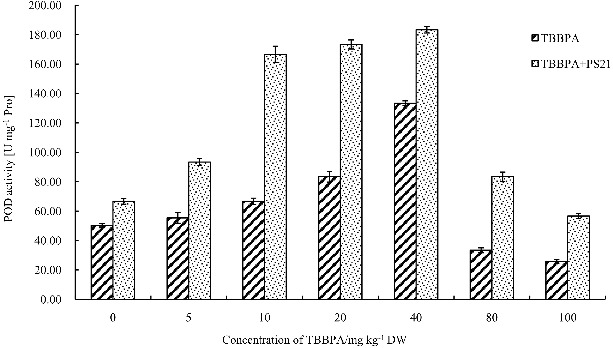
Changes of the POD activity in wheat leaves with combined treatment of PS21 and different concentrations of TBBPA. Values are mean ± SD and bars indicate standard deviation. TBBPA: treatment with various concentrations TBBPA (0–100 mg kg^−1^ DW); TBBPA + PS21: with combined treatment with PS21 and various concentrations TBBPA (0–100 mg kg^−1^ DW).

SOD, CAT and POD are the main plant antioxidant enzymes and can remove active oxygen radicals in order to protect plant cells.[38] SOD catalyzes the dismutation of O_2_^−^ into H_2_O_2_ and O_2_, CAT and POD catalyze the decomposition of H_2_O_2_ to H_2_O and O_2_. SOD, CAT and POD levels rise upon stress to remove reactive oxygen species (ROSs) and improve the stress resistance of plants. In order to analyse the damage on wheat seedlings caused by TBBPA, antioxidant enzymes activities (POD, SOD and CAT) of wheat seedlings were measured. Low concentrations of TBBPA treatment resulted in a significant increase in SOD, POD and CAT activities of wheat seedlings as compared to the control (Figures 3–5). At 20 mg kg^−1^, the SOD and CAT activities of wheat seedlings reached the maximum level, increasing by 202.22% and 230.88% compared to the control, respectively (*P* < 0.01) (Figures 3 and 4), while for POD, the highest activities appeared at the TBBPA concentration of 40 mg kg^−1^, increasing by 164.91% (Figure 5). However, SOD, POD and CAT activities declined with further increasing the concentration of TBBPA. These results suggested that high concentrations of TBBPA inhibit SOD, POD and CAT activities.

As can be seen from Figure 3, a concentration of 5--40 mg kg^−1^ TBBPA can significantly increase the SOD activity of wheat seedlings as compared to control (*P* < 0.05). The seedlings treated with 80 and 100 mg kg^−1^ TBBPA showed no significant increase in SOD enzyme activity compared to control (*P* > 0.05) suggesting that higher concentrations of TBBPA inhibited the enzyme activity. The treatment of PS21 increased the SOD activity of wheat seedlings treated with 5--100 mg kg^−1^ TBBPA to varying degrees. SOD activity was increased by 6.67%, 21.45%, 2.94%, 15.43%, 26.13% and 59.39% in response to PS21 in wheat seedlings treated with 5, 10, 20, 40, 80 and 100 mg kg^−1^ TBBPA, respectively (*P* < 0.05). PS21 showed the best effect on wheat seedlings treated with 100 mg kg^−1^ in terms of SOD activity, and the activity was increased by 59.39%. It is suggested that the composite inoculant PS21 can increase the capacity of the plant to cope with stress, and therefore, relieve the damage caused by TBBPA.

AS showed in Figure 4, a concentration of 5–80 mgkg^−1^ TBBPA can significantly increase the CAT activity of wheat seedlings as compared to control (*P* < 0.05). However, a concentration of 100 mg kg^−1^ TBBPA did not significantly increase the CAT activity in wheat seedlings (*P* > 0.05). The treatment of PS21 significantly increased the CAT activity of the wheat seedlings by 123.36%, 153.93%, 72.62%, 38.17%, 34.87% and 117% when treated with 5, 10, 20, 40, 80 and 100 mg kg^−1^ TBBPA (*P* < 0.01), respectively. PS21 showed the best detoxification effect on wheat seedlings treated with 10 mg kg^−1^ TBBPA, leading to the highest increase in CAT activity.

Wheat seedling groups treated with 5–40 mg kg^−1^ TBBPA showed a significant increase in POD activity compared to the control (*P* < 0.05) (Figure 5). However, wheat seedlings treated with 80 and 100 mg kg^−1^ TBBPA showed lower POD activity, which decreased by 33.78% and 49.08%, respectively. The treatment of PS21 can significantly increase POD activity of the wheat seedlings treated with 5, 10, 20, 40, 80 and 100 mg kg^−1^ TBBPA (*P* < 0.01), and POD activity was increased by 68.68%, 149.99%, 108%, 37.50%, 149.99% and 121.10%, respectively. PS21 showed the best detoxification effect on the wheat seedlings treated with 10 and 80 mg kg^−1^ TBBPA, which led to the highest increase in POD activity.

Composite photosynthetic bacterium inoculants PS21 can increase SOD, CAT and POD activity of the wheat seedlings under TBBPA stress, which might be that photosynthetic bacteria can degrade organics and produce a variety of B vitamins including pantothenic acid, amino acids, coenzyme Q, 5-aminolevulinic acid (ALA), porphyrin compounds, plant hormones (such as IAA, GA3, ethylene and cytokinins) and antiviral factors.[21–24] These substances can stimulate the activity of plant cells improving the efficiency of photosynthesis as well as the activities of SOD, CAT and POD to enhance the resistance of plants to stress. Lee et al. [26] reported that *Rhodopseudomonas* sp. KL9 and BL6 strains can produce IAA and ALA. Gossett et al. [39] reported that IAA can increase the antioxidant enzyme activity to protect plant cells from oxidative damage and to regulate the metabolism of plants. Our results corroborate these studies. However, in these studies, a large amount of bacteria was required and the effects on plant growth were mild due to the use of single strain, whereas in our study, two stains were used to generate the composite inoculants that have a synergistic effect and can secrete active substances to improve SOD, CAT and POD activity. Our study found that the composite photosynthetic bacterium inoculants PS21 had a strong detoxification effect on TBBPA, which could be attributed to two reasons: (1) PS21 were capable of degrading TBBPA; (2) PS21 produced growth-promoting factors (ALA, porphyrin compounds, plant hormones and antiviral factors) to enhance stress resistance and growth of plant.

### The effects of combined PS21 and TBBPA treatment on soluble protein content of wheat seedlings

**Figure 6.**
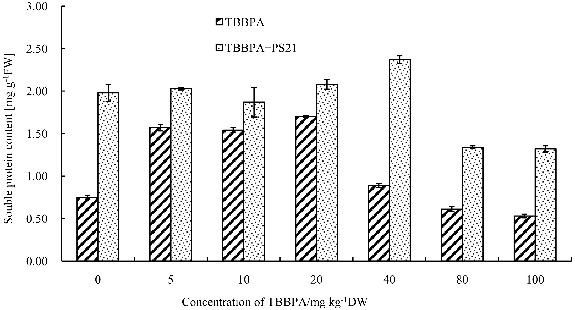
Changes of the soluble protein content in wheat leaves with combined treatment of PS21 and different concentrations of TBBPA. Values are mean ± SD and bars indicate standard deviation. TBBPA: treatment with various concentrations TBBPA (0–100 mg kg^−1^ DW); TBBPA + PS21: with combined treatment with PS21 and various concentrations TBBPA (0–100 mg kg^−1^ DW).

Soluble proteins are an important part of plant active substances, and plants often accumulate some soluble proteins to induce antioxidant enzymes, detoxification enzymes, metabolic enzymes and some function factors for eliminating free radicals in plants under various stresses.[40] Therefore, the accumulation of soluble proteins can also be considered as a self-protective response against TBBPA. Our results showed that the increasing of TBBPA concentration resulted in improving the soluble protein content of wheat seedlings at first stage, and then followed by a decrease in the content (Figure 6). The soluble protein content reached the highest level when the TBBPA concentration was 20 mg kg^−1^ (increased by 126.67% compared to the control). Statistical analysis showed that 5--100 mg kg^−1^ TBBPA led to significant changes in soluble protein content compared to the control (*P* < 0.05). Soluble protein content showed a marked decrease when the wheat seedlings treated with 80--100 mg kg^−1^ TBBPA, and reduced by 18.67% and 29.33%, respectively. Our results were in accordance with the findings of Shang et al. [40]. It could be that certain concentrations of TBBPA caused soluble protein accumulation in wheat seedlings to keep the balance of water in plant cell and tissues, stabilize the biological macromolecular structure.[40] However, soluble protein content of wheat seedlings treated with TBBPA was significantly enhanced after inoculating PS21 (*P* < 0.01), which suggested that PS21 significantly alleviated the inhibition of soluble protein synthesis caused by TBBPA. But the favourable effects had no significant correlation with TBBPA concentration. PS21 had best effects on wheat seedlings treated with 40 mg kg^−1^ TBBPA in terms of soluble protein content, which was increased by 166.29% in comparison to that of the seedlings only treated with 40 mg kg^−1^ TBBPA. Thus, the composite photosynthetic bacterium inoculants can alleviate the damage of TBBPA on wheat seedlings and enhance the resistance of wheat seedlings to TBBPA by improving soluble protein content.

### The effect of combined PS21 and TBBPA treatment on MDA content of wheat seedlings

**Figure 7.**
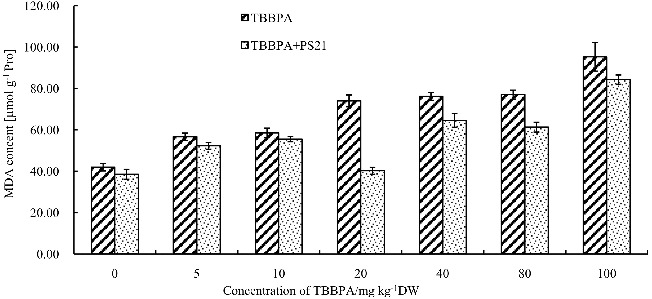
Changes of MDA levels in wheat leaves with combined treatment of PS21 and different concentrations of TBBPA. Values are mean ± SD and bars indicate standard deviation. TBBPA: treatment with various concentrations TBBPA (0–100 mg kg^−1^ DW); TBBPA + PS21: with combined treatment with PS21 and various concentrations TBBPA (0–100 mg kg^−1^ DW).

MDA is an important product of membrane lipid peroxidation, and MDA content is one of the indicators used to assess plant cell membrane damage. Too much ROS can also inhibit SOD, CAT and POD activities, leading to an increase in the MDA content and cell damage. In order to analyse whether PS21 treatment could decrease the damage on cell membrane caused by TBBPA, MDA content was measured after PS21 treatment. At present, MDA content of wheat seedlings showed an increasing trend with increase in TBBPA concentration (Figure 7). The increase in each concentration was significantly different compared to the control (*P* < 0.05). The highest MDA content was observed in seedlings treated with 100 mg kg^−1^ TBBPA showing a 127.16% increase compared to the control. In this study, the increase in MDA indicated that TBBPA treatment led to oxidative stress. Moreover, as the TBBPA concentration increased, the MDA content also increased, indicating that the generation of ROSs, as well as the damage by TBBPA on wheat seedlings were enhanced, which is consistent with previous studies.[20] After the treatment of PS21, the MDA content in wheat seedlings of each treatment group decreased significantly (*P* < 0.01). PS21 and 20 mg kg^−1^ TBBPA together resulted in the lowest MDA content in wheat seedlings as compared to other treatments and the control. Moreover, the MDA content of the abovementioned treatment was not significantly different from that of seedlings treat with PS21 alone (*P* > 0.05). Thus, the composite photosynthetic bacterium inoculants alleviated the damage on wheat seedling caused by TBBPA.

## Conclusion

In sum, PS21 can mitigate the damage inflicted on wheat seedlings by various concentrations of TBBPA. We discussed the physiology mechanism by measuring total chlorophyll content, soluble sugars content, soluble protein content and activities of antioxidant enzymes, as well as MDA content. The results showed that sugars content, soluble protein content, total chlorophyll content and activities of antioxidant enzymes increase and further lead to the decreasing of MDA content. Meanwhile, we found that the effectiveness depends on the PS21 concentration and the methods (data not shown) of application. Owing to the interactions of plants, PS21 and TBBPA have a complex action. Therefore, we need to do further work to investigate a deeper mechanism.

## Disclosure statement

The authors declare that there is no conflict of interest.

## Funding

This study was funded by Science and Technology Key Project of Educational Commission of Henan Province, China [grant number 13B180271], [grant number 12B210028]; Science and Technology Planning Project of Science and Technology Commission of Henan Province, China [grant number 132300410032].
